# What drives Chinese youth to use fitness-related health information on social media? An analysis of intrinsic needs, social media algorithms, and source credibility

**DOI:** 10.3389/fpubh.2024.1445778

**Published:** 2024-12-05

**Authors:** Xin Zhang, Qing Qing Tang, Ying Ying Cai

**Affiliations:** Department of Communication, Faculty of Modern Languages and Communication, Universiti Putra Malaysia, UPM Serdang, Selangor, Malaysia

**Keywords:** youth, social media, fitness-related health information, intrinsic needs, social media algorithms, source credibility

## Abstract

**Introduction:**

The role of social media in providing fitness-related health information has been widely discussed; however, there is a notable lack of research on fitness-related health information behaviors among youth within the social media context. This study aims to address this gap by integrating Self-Determination Theory (SDT)-based internal factors and external factors (social media algorithms and source credibility).

**Methods:**

A voluntary sample of 600 participants, aged 15 to 29, was recruited. Data were analyzed using Partial Least Squares-Structural Equation Modeling (PLS-SEM) to examine the relationships between variables.

**Results:**

The analysis revealed that all three intrinsic needs—competence, autonomy, and relatedness—along with social media algorithms and source credibility, positively correlated with fitness-related health information use behaviors among youth. Additionally, social media algorithms moderated the relationship between the need for relatedness and fitness-related health information behavior.

**Discussion:**

These findings provide new insights into developing health communication strategies on social media, particularly targeted toward the youth demographic, enhancing our understanding of effective health information dissemination in digital environments.

## 1 Introduction

### 1.1 Social media’s impact on health education and fitness information

Existing research has highlighted the positive impact of social media in the health domain (1), largely due to its pervasive influence. Social media holds significant potential in advancing health education and promotion activities, such as engaging broad audiences in social marketing campaigns and enhancing consumer interactions in health and healthcare (2). Additionally, scholars have examined the health behaviors of various groups on social media, including the older adult (3), youth (4), women (5), and sexual minorities (6).

Given the diverse knowledge encompassed by health information, it is necessary to conduct targeted studies on specific groups’ urgent health information needs to help them change harmful habits and behaviors. These studies could focus on topics such as cancer, oral health, mental health, and fitness-related health.

Over the past 50 years, global obesity rates have steadily increased, reaching epidemic levels, especially in the Asia-Pacific region. Reducing the health and social burdens associated with obesity and reversing its rising prevalence is a top priority for the World Health Organization (7). Increasing physical activity is a primary intervention (8). Consequently, citizens, governments, and health education organizations are increasingly focusing on the dissemination of fitness-related health information via social media.

### 1.2 The potential of social media for fitness-related information among youth

In various cultural and research contexts, individuals under the age of 29 are often included in the category of “youth” (9, 10). The health and physical fitness of youth are crucial not only for the individuals but also for society as a whole (11). However, research indicates that youth often lack physical exercise, with this trend worsening with age. Additionally, women are significantly less active than their male counterparts (12). A qualitative study in China found that young people have limited knowledge about health and fitness, and some are indifferent to their health (13).

To increase overall physical activity, Choi and Jiang (14) found that enhancing access to and sharing of fitness-related information can help boost exercise levels. For youth, the sources of health information have shifted from traditional health education classes to online platforms (15). This demographic prefers to obtain health-related information from social media (16–19). Social media holds significant potential for disseminating fitness and health information (20). Indeed, social media has transformed the dissemination of fitness-related health information. For example, the #fitspiration tag on Instagram has gained widespread popularity, becoming a major source of motivation for users to pursue fitness (21). Similarly, video platforms like YouTube have facilitated the spread of fitness knowledge and encouraged user engagement (22). Additionally, many users tend to trust content posted by verified accounts or influential fitness influencers (23).

However, research on youth’ use of fitness-related health information on social media is still limited. Current studies primarily focus on online search behaviors for fitness health information and fitness activities on social media (16, 17, 20). Also, Existing research primarily focuses on user behavior in Western countries, with limited understanding of the motivations and behaviors of young people in China regarding their use of fitness-related information on social media (24, 25).

### 1.3 Fitness-related health communication on Chinese social media

Social media primarily refers to “Web 3.0 social media,” including platforms like Twitter, Instagram, TikTok, Weibo, WeChat, as well as collaborative wikis, blogs, and mobile platforms that connect people through interactive messaging and digital assistants (2).

Globally, fitness-related health communication is highly active on social media (26). In the United States, social media promotes healthy behaviors, with young users often inspired by shared fitness achievements and healthy eating posts (27). In Europe, interactions like likes and comments enhance exercise motivation (28). In Southeast Asia, especially on Facebook, social media serves as a key tool for government health promotion (29). In Hong Kong, social media influencers significantly impact young people’s diet and fitness behaviors (30). In Mainland China, social media platforms like WeChat, TikTok, and Weibo are integral to daily life. Fitness live streaming is especially popular, fostering positive attitudes, reducing costs, and building fitness communities among users (31).

Furthermore, surveys of young people in China have shown that fitness apps with social media features (such as “KEEP,” which supports community interactions, private messaging, health information dissemination, and fitness live streaming) can effectively promote a healthy lifestyle among the youth (32). Given the large number of young social media users in China (33), this environment is suitable for studying how youth use fitness-related health information on social media.

### 1.4 Integrating SDT and external factors in studying fitness-related health information on social media

Using social media is often considered a self-motivating behavior (34), with self-determination being a prominent feature of social media use (35). However, current research on fitness-related health information primarily focuses on external factors, such as information verification, resources, parental influence, and barriers to information access (17), lacking empirical studies that consider user behavior from the perspective of individual psychological needs.

To address this gap, the study incorporates SDT (36) to explain individuals’ behavior in using fitness health information on social media from the aspect of intrinsic needs. According to SDT, individuals have three intrinsic needs: competence (reflecting the desire for mastery and efficacy), autonomy (reflecting the desire for self-initiation and self-regulation), and relatedness (reflecting the desire for connection with others) (35). Theories such as the Health Belief Model (HBM), Theory of Planned Behavior (TPB), and Technology Acceptance Model (TAM) also offer valuable insights into health information behavior. HBM focuses on perceived health threats (37), TPB on subjective norms (38), and TAM on ease of use and usefulness (39). However, these theories emphasize extrinsic motivation and social norms, offering limited insight into autonomy and intrinsic motivation. In contrast, SDT emphasizes satisfying intrinsic needs, essential for sustaining behavior (40).

While considering personal intrinsic needs is essential, certain external environmental factors also significantly influence individuals’ behavior in using fitness health information on social media. Thus, the researchers aim to develop a model that integrates external factors alongside individual needs. On one hand, information resources and verification are crucial for individuals’ use of fitness health information (17), as is the source of the health information (18). On the other hand, with the advancement of social media algorithms, people might be influenced by algorithm-assigned tasks without being aware of them (41), making it necessary to consider the impact of social media algorithms on users. Currently, empirical studies on understanding algorithms from the user’s perspective are quite limited (42).

Based on the aforementioned research gaps, the study considers two external factors, integrating them with individual psychological needs to examine the use behavior of fitness health information by Youth on social media. The research posits three questions:

- what SDT intrinsic factors influence youth’ behavior in using fitness-related health information on social media?
- Do social media algorithms and source credibility influence youth’ behavior in using fitness-related health information on social media?
- Do social media algorithms have a moderating role between SDT intrinsic factors and youth’ behavior in using fitness-related health information on social media?

Theoretically, these results help explain how psychological needs, social media algorithms, and content credibility influence young people’s use of fitness health information on social media. Practically, this can guide health educators and information providers in China and beyond to refine communication strategies targeting young audiences on social media.

## 2 Theoretical foundation

### 2.1 SDT and social media

SDT originated from a study on the impact of external rewards on intrinsic motivation (43) and was later formally proposed by Deci and Ryan. It is a macro-theory in psychology concerning individual motivation and human behavior (44). SDT emphasizes the intrinsic motivation and basic psychological needs underlying human behavior, with core concepts including the needs for autonomy, competence, and relatedness (45). The need for autonomy refers to the desire to feel in control of one’s actions and decisions (45); the need for competence involves the desire to feel effective in interactions with the environment and to master challenges (46); and the need for relatedness refers to the desire to feel connected to others and to have a sense of belonging, including close relationships, social connections, and perceived social support (45). SDT posits that fulfilling these basic psychological needs promotes greater engagement, motivation, and satisfaction in activities (47).

SDT distinguishes between intrinsic motivation and extrinsic motivation, with extrinsic motivation being further divided into external regulation, introjected regulation, identified regulation, and integrated regulation (45). While extrinsic motivation has been extensively discussed about similar research variables, such as subjective norms, perceived behavioral control, and the ease of use and usefulness of new technologies (38, 39, 48), users in social media environments exhibit their own will and needs (35). Individuals using social media often express strong needs for autonomy and personalization (49). Therefore, focusing on personal intrinsic needs is crucial when researching social media, and this study emphasizes intrinsic motivation exclusively.

Existing research demonstrates the suitability of SDT for studies related to social media. For instance, understanding the sustained use of health communities from a self-determination perspective reveals that SDT’s intrinsic motivation can aid in community management and system design, thereby promoting continuous user engagement (50). Additionally, fulfilling SDT’s intrinsic motivations can enhance user participation and electronic word-of-mouth on social networking sites (51). In studies focusing on employee social media use, it has been found that the need for competence, autonomy, and relatedness influences employee motivation to use social media in various contexts (35).

### 2.2 SDT and fitness-related health information use behavior

The application of SDT is extensive, encompassing fields such as education, work, sports, and health (52). In the realm of sports and health, existing research primarily focuses on the exercise motivations, exercise beliefs, exercise roles, and body image of adolescents and adults (53–55). These studies consistently indicate a positive correlation between more autonomous forms of motivation and exercise, where satisfaction of competence and intrinsic motivation significantly predict exercise participation across various samples and settings, making SDT particularly suitable for research in the domain of physical exercise (54).

Data from a survey of 350 employees at three large teaching hospitals in Taiwan indicate that intrinsic motivation plays a critical role in the knowledge use and sharing processes within health information systems (56). A meta-analysis by Gillison et al. (57) of 74 articles on techniques to promote motivation for health behavior change found that changes in health behavior require the combined use of health information and the fulfillment of self-determined needs, suggesting an intrinsic link between health information use and self-determined needs. Furthermore, Ng et al. (58) conducted a meta-analysis of 184 articles using SDT in the context of healthcare and health promotion, revealing a positive correlation between the satisfaction of psychological needs, intrinsic motivation, and beneficial health outcomes. This demonstrates that SDT is a viable conceptual framework for studying health-related behaviors.

Based on the above discussion, using SDT as a theoretical framework to study fitness-related health information use behavior is justified.

### 2.3 Fitness-related health information use behavior

Health information use behavior involves the processes of acquiring, understanding, evaluating, and applying health information (59, 60). In this study, we focus on the behavior of Chinese youth using fitness-related health information through social media. These behaviors include information acquisition, understanding, evaluation, and application (61, 62).

Specifically, information acquisition refers to obtaining relevant fitness and health information through social media platforms such as WeChat, Weibo, Instagram, TikTok, and others. Information understanding involves comprehending the content of fitness information on social media, such as proper exercise methods and nutritional advice. Information evaluation pertains to assessing the credibility of the information, such as determining whether the advice from a fitness influencer or medical professional is trustworthy. Information application refers to the practical use of this information to engage in fitness activities or change lifestyle habits.

## 3 Conceptual framework

**Figure 1:**
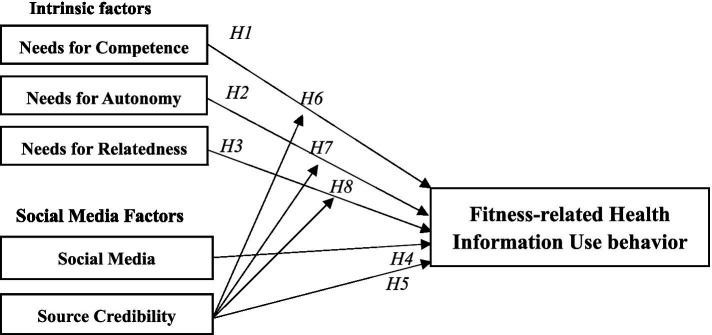
Conceptual framework.

The study incorporated three types of individual intrinsic needs—competence, autonomy, and relatedness—along with external factors (social media algorithms and source credibility) to investigate the factors influencing the motivation and behavior for using fitness-related health information on social media. Furthermore, the research examined the moderating role of social media algorithms between intrinsic needs and use behavior. A detailed framework diagram is shown in Figure 1.

### 3.1 Needs for competence

From the Need-Affordability-Functionality (NAF) perspective, an individual’s psychological needs prompt them to use social media applications, with competence needs playing a critical role when the availability of individual needs is met (34). When an individual’s competence needs are satisfied, it enhances the likelihood of user-generated content (63). With challenging goals, i.e., when competence needs are low, users may not specifically focus on the design of social media. Conversely, when individuals have higher goals, or a greater sense of competence, they are more likely to be motivated (64). Studies on Millennials’ use of social media have also found that the more competent they feel, the more they engage with social media (65). When it comes to fitness-related health information, the more an application satisfies individual competence needs, the higher the likelihood of motivating consumers, and the more likely consumers are to engage and consume (66). Therefore, the following hypothesis is proposed:

*H1:* Needs for competence is positively related to fitness-related health information use behavior on social media among youth.

### 3.2 Needs for autonomy

Needs for Autonomy significantly influence individual health motivation and behavior on social media, especially in the health domain (67). For fitness health information on social media platforms, there is tremendous potential, such as helping young people build emotional communities (19). Particularly, female groups in fitness communities experience more autonomy, and viewing “fitness inspiration” images may promote their further information use behavior (68), despite some negative effects of using social media for fitness information (69). High levels of autonomous intrinsic motivation may involve exercising for important personal health goals (identified regulation) or, at the most intrinsic level, exercising becomes an integral part of the self, aiding in achieving positive well-being and practicing long-term information acceptance and exercise behavior (47, 70). Thus, the following hypothesis is proposed:

*H2:* Needs for Autonomy is positively related to fitness-related health information use behavior on social media among youth.

### 3.3 Needs for relatedness

Needs for Relatedness have been proven to positively impact health information search behavior in previous health behavior studies (71). Additionally, in studies on fitness-related health information behavior, social acceptability, confidence, family and friends’ pressure can influence the behavior related to fitness health information among college students (17). Specifically, after individuals share fitness-related health information, it involves details about fitness goals, achievements, and challenges, which may help garner social support, thereby promoting further use of such information on social media (72). Secondly, relatedness needs might drive individuals to share personal health achievements, workout plans, and physical changes on social media to establish resonance and comparison (73). Finally, users may lean toward social identification with fitness-related information or perspectives within the framework of relatedness needs, conforming to the expectations of social groups (74). Therefore, the following hypothesis is proposed:

*H3:* Needs for Relatedness is positively related to fitness-related health information use behavior on social media among youth.

### 3.4 Social media algorithms

Algorithms increasingly influence how young people perceive the world around them (75). Social media platforms use algorithms to customize users’ content experiences, which can impact their motivation and behavior (76). By analyzing users’ behavior, preferences, and interaction history, algorithms provide personalized health information, which may inspire individuals to engage more with health topics relevant to their personal interests (77). Moreover, algorithms use incentive and reward mechanisms, such as feedback from likes, shares, and comments, to increase user engagement (78, 79), influencing their motivation to actively participate in health information interactions. Thus, the following hypotheses are proposed:

*H4:* Social Media Algorithms is positively related to fitness-related health information use behavior on social media among youth.

### 3.5 Source credibility

Source credibility refers to the ability or motivation of an information source to provide accurate and truthful information (80), and it can influence users’ motivation and behavior in using fitness-related health information from multiple perspectives. Firstly, from the publishers’ perspective, trusted, expert, and attractive social media fitness influencers can effectively increase users’ fitness intentions and behaviors (81). Secondly, the quality of the content itself influences users’ motivation to use fitness-related health information. When users verify the information (for example, seeking validation from doctors and experts in the field or checking against books), it further impacts their behavior in using fitness-related health information (17). Therefore, the following hypothesis is proposed:

*H5*: Source credibility is positively related to fitness-related health information use behavior on social media among youth.

### 3.6 Moderating role of social media algorithms between the need for competence, autonomy and relatedness and fitness-related health information use behavior

Algorithms can have two distinct effects on autonomous behavioral outcomes: on one hand, they allow users to autonomously define themselves, but on the other hand, they can threaten users’ choices and freedom (82). In the technological world, algorithms operate within an opaque framework, inadvertently reshaping users’ values through the information they present (83). The suppressive effect of algorithmic technology on the concept of self-directed action suggests that it is increasingly challenging to maintain a clear sense of self-determination (84). For instance, fitness devices connected to social media can lead users to reasonably disregard their actions through algorithms, affecting their self-assessment of their abilities (85). Therefore, it is necessary to examine the role of social media algorithms in the relationship between competence, autonomy needs, and behavior.

Furthermore, research indicates that the filter bubbles created by social media algorithms can impact the number of friends users follow (86), and subtly control users’ perception and sharing behaviors (87). These factors can influence users’ relatedness needs. Consequently, the following hypotheses are proposed:

*H6:* Social media algorithms play a moderating role between needs for competence and fitness-related health information use behavior on social media among youth.

*H7:* Social media algorithms play a moderating role between needs for autonomy and fitness-related health information use behavior on social media among youth.

*H8:* Social media algorithms play a moderating role between needs for relatedness and fitness-related health information use behavior on social media among youth.

## 4 Research methods

### 4.1 Study design

This study employed a cross-sectional design, collecting data via an online questionnaire from January 10, 2024, to April 30, 2024, to examine the use behaviors of fitness-related health information among youth in China. The sample included individuals aged 15 to 29 who either had experience using social media for fitness-related health information or expressed potential interest in such content.

### 4.2 Measurement

The survey comprises two sections. Part A collects basic demographic information, including age, income, marital status, education level, preferred social media for fitness-related information, and experience with these platforms. Part B uses a seven-point Likert scale (“strongly disagree” to “strongly agree”) to examine factors influencing fitness-related health information use.

For Part B, Intrinsic Factors in this study are adapted from the scope of research on SDT by Demircioglu (88) and Wei, Chen and Liu (35). Specifically, Needs for Competence are explored through five dimensions: mastery of skills and knowledge, enhancement of overall ability, increase in experience, tendency to use, and proactive usage. Needs for Autonomy are addressed through items crafted around the self-determination of usage time, location, recommendations, access platforms, and autonomy in expression and practice. Needs for Relatedness are adapted from the needs for interaction with others, the degree of interaction, and social support.

In terms of social media algorithm dimensions, research indicates that settings of recommendation algorithms (89), filtering algorithms (90), personalization (91), algorithm transparency (76), and feedback mechanisms (92) affect user engagement on social media. Thus, this study employs these five dimensions to measure the motivations and behaviors of users regarding the use of fitness-related health information. Moreover, source credibility in the study is primarily measured through safety qualification, dynamism, and sociability, following the definition and assessment criteria established by Berlo et al. (93). Finally, the study of behavior in using fitness-related health information is adapted (50) and developed based on four dimensions: duration, search themes, scope, and number of participants (94). See Supplementary Appendix B for specific items.

A pilot study was conducted before the formal questionnaire release. We began with a content validity test, where two experts in social media and health communication reviewed the questionnaire, providing feedback on each question’s relevance and clarity. After incorporating their suggestions, we revised the questionnaire to better capture the core constructs. We then conducted the pilot study with 63 participants similar to our formal study group, assessing the reliability of each scale. All constructs showed high reliability (95), as detailed in Table 1. Based on participant feedback and data analysis, we revised 10 unclear questions before launching the formal study.

**Table 1:** Reliability results for pilot study.

Constructs	Number of Items	Cronbach’s α
Needs for autonomy	5	0.914
Needs for competence	5	0.879
Needs for relatedness	6	0.895
Social media algorithms	5	0.92
Source credibility	5	0.88
Fitness-related health information use behavior	6	0.9

### 4.3 Sample calculation and sampling method

For PLS-SEM data analysis, the recommended sample size should be at least 10 times the number of formative indicators in the largest scale (96), meaning a minimum of 60 if the largest indicator count is 6. Additionally, using Israel’s (97) sample size formula *N* ≥ 20 × *k* / (1 - *R*^2^), where k is the number of latent variables and *R*^2^ represents the strongest relationship in the model (typically between 0.1 and 0.5), a mid-value of *R*^2^ = 0.3 yields a required minimum of approximately 172. Given the model’s complexity and anticipated effect size, a final sample size of 600 valid responses was chosen.

To effectively reach young people using fitness-related health information, voluntary response sampling was conducted in mainland China. This approach attracted individuals with high interest in fitness and social media, improving data relevance. However, this method may limit sample representativeness by excluding low active users and skewing the sample toward younger, tech-savvy individuals, potentially impacting generalizability.

### 4.4 Data collection procedure

Data collection was conducted from January 10, 2024, to April 30, 2024, through several targeted recruitment channels. On the WJX.cn platform, we utilized a point-based community system to recruit participants. The distributor’s points will be deducted for each questionnaire collected. Specific eligibility criteria were set to ensure that only individuals meeting the study’s requirements could participate. As China’s largest free survey platform, WJX.cn can reach approximately 300 million monthly users (98, 99) and 1.51 million daily active users (100), making it an effective channel for survey distribution. In addition to WJX.cn, we also recruited participants through WeChat groups and Tencent QQ groups. Fitness enthusiast communities on WeChat provided access to individuals likely to be interested in the study topic, enhancing sample relevance. Similarly, on Tencent QQ, we targeted university student survey groups to attract youth with an interest in fitness-related health information.

A total of 600 valid responses were collected after excluding 60 invalid responses, identified by response times under 1 min or illogical answers, resulting in a 90% response rate. Participants accessed the survey via a link on their PC or mobile device and received a 1 RMB incentive upon completion. Each participant reviewed and approved an informed consent form before beginning the survey, and the questionnaire was translated from English to Chinese to ensure clarity and comprehension for all respondents (101).

### 4.5 Data analysis

Data analysis used SPSS 25.0 for demographic data and SmartPLS 4.0 for motivational factors through PLS-SEM, which is effective for small, non-normal samples and exploratory research (102). While CB-SEM is favored for confirmatory studies with well-defined models, it requires normal distribution and a larger sample size (103). Thus, PLS-SEM was chosen for its adaptability.

## 5 Statistical analysis and results

### 5.1 Demographic characteristics, social media platform preference, and experience of respondents

The survey participants were primarily youth, with an average age of approximately 22.83 years, predominantly ranging from 15 to 29 years old. A significant majority of the participants were female, accounting for 71.83%. In terms of educational attainment, most respondents (79.33%) held a bachelor’s degree, followed by 11% who possessed master’s degrees. Regarding marital status, the majority of participants were single (73.17%), with a small portion in a romantic relationship. In terms of income, the vast majority of respondents reported a monthly income between 1,001 to 3,000 yuan. Detailed data are shown in Table 2.

**Table 2:** Demographic characteristics of participants.

Characteristic	Frequency	Percentage (%)
Age (*M* = 22.80, SD = 2.54)		
**Male**	169	28.17
**Female**	431	71.83
Education level		
High school/Junior college	10	1.67
College	40	6.67
Undergraduate	476	79.33
Master’s degree	66	11
Ph.D.	8	1.33
Marital status		
Single	439	73.17
Married	40	6.67
In a loving relationship	121	20.17
Income level		
1,000 RMB and below	123	20.50
1,001–3,000 RMB	261	43.50
3,001–5,000 RMB	93	15.50
5,001–10,000 RMB	85	14.17
10,001–15,000 RMB	25	4.17
15,001–20,000 RMB	8	1.33
20,001 and above	5	0.83

Regarding preferences for social media platforms for accessing fitness-related health information, TikTok emerged as the most popular platform, followed by Xiaohongshu (Red). TikTok accounted for 33.17% of the preference, with Red closely following at 31.00%. WeChat and Weibo were less favored, with only 13.00 and 5.17% of users choosing them, respectively. From the results regarding the experience of users in accessing fitness-related health information on social media, those who have been using these platforms for a short term (less than 6 months) constituted the highest proportion at 57.34%. This indicates that most users are relatively new to accessing this type of information. Longer-term users (1–3 years and 3–6 years) were less common, accounting for only 17.33 and 6.50%, respectively. This might suggest that users tend to decrease the frequency of searching for health information on social media over time. Detailed data are shown in Tables 3, 4.

**Table 3:** Social media platform preferences for fitness-related health information.

Social media platform	Frequency	Proportion (%)	Notes
WeChat	78	13.00	Preferred platform for health information
Weibo	31	5.17	Similar to Twitter
TikTok (Douyin)	199	33.17	Most popular platform
Kuaishou	11	1.83	Similar to TikTok
Red (Xiaohongshu)	186	31.00	Second most popular platform
Reddit, Quora	26	4.33	For international users
Fitness APP	68	11.33	Specialized health apps
Instagram, Facebook, or Twitter	1	0.17	Least preferred

**Table 4:** Experience using social media for fitness-related health information.

Experience	Frequency	Proportion (%)	Notes
Less than 1 month	163	27.17	Initial exposure to health content on social media
Less than 6 months	181	30.17	Most Selected Options
6–12 months	113	18.83	/
1–3 years	104	17.33	Moderate long-term use
3–6 years	39	6.50	Long-term experienced users

### 5.2 Construct reliability and validity analysis

In this study, ensuring the reliability and validity of measurement instruments is paramount. Reliability is verified through Cronbach’s alpha (rho_a) and composite reliability (rho_c), with all values surpassing the 0.7 threshold, indicating high internal consistency of the constructs (104). This confirms that the constructs reliably measure the intended variables. Convergent validity, assessed via Average Variance Extracted (AVE) and outer loadings, is also sufficiently demonstrated. All constructs display AVE values exceeding the 0.5 standard and show outer loadings above 0.7 (Table 5), confirming that the constructs adequately capture the variance within their indicators and that the items are strongly correlated within each construct (105). These metrics ensure the constructs’ ability to provide precise and reliable measurements, bolstering the study’s statistical integrity.

**Table 5:** Construct reliability and validity measures.

Constructs	Cronbach’s α	Composite reliability (rho_a)	Composite reliability (rho_c)	AVE
Fitness-related health Information use behavior	0.827	0.832	0.874	0.537
Needs for autonomy	0.832	0.832	0.881	0.598
Needs for competence	0.836	0.839	0.883	0.603
Needs for relatedness	0.884	0.898	0.914	0.681
Social media algorithms	0.859	0.868	0.897	0.636
Source credibility	0.834	0.835	0.883	0.602

### 5.3 Discriminant validity analysis using Fornell-Larcker criterion and HTMT

Discriminant validity ensures that constructs within a model are unique and not overly similar to one another. The Fornell-Larcker criterion requires that the square root of the Average Variance Extracted (AVE) for each construct should exceed its highest correlation with any other construct, demonstrating that constructs share more variance with their indicators than with other constructs (105). The Heterotrait-Monotrait (HTMT) ratio, as another measure, should be below 0.90 to confirm that constructs are more similar within than between them (106). In the study, both criteria are met: the square roots of AVEs are higher than the correlations between constructs, and all HTMT values are below 0.90 (Tables 6, 7). This indicates robust discriminant validity, showing that each construct distinctly measures specific aspects of the model without significant overlap with others, thereby supporting the accuracy and integrity of the model’s structure.

**Table 6:** Fornell-Larcker testing.

	Fitness-related health information use behavior	Needs for autonomy	Needs for competence	Needs for relatedness	Social media algorithms	Source credibility
Fitness-related health information use behavior	0.733					
Needs for autonomy	0.584	0.774				
Needs for competence	0.609	0.675	0.776			
Needs for relatedness	0.376	0.244	0.374	0.825		
Social media algorithms	−0.172	−0.132	−0.097	−0.026	0.798	
Source credibility	0.698	0.625	0.574	0.348	−0.138	0.776

**Table 7:** Heterotrait-Monotrait ratios (HTMT).

**Factors**	**HTMT ratio**
Needs for autonomy ↔ Fitness-related health information use behavior	0.7
Needs for competence ↔ Fitness-related health information use behavior	0.725
Needs for competence ↔ Needs for autonomy	0.81
Needs for relatedness ↔ Fitness-related health information use behavior	0.427
Needs for relatedness ↔ Needs for autonomy	0.268
Needs for relatedness ↔ Needs for competence	0.422
Social media algorithms ↔ Fitness-related health information use behavior	0.198
Social media algorithms ↔ Needs for autonomy	0.154
Social media algorithms ↔ Needs for competence	0.113
Social media algorithms ↔ Needs for relatedness	0.069
Source credibility ↔ Fitness-related health information use behavior	0.837
Source credibility ↔ Needs for autonomy	0.748
Source credibility ↔ Needs for competence	0.687
Source credibility ↔ Needs for relatedness	0.395
Source credibility ↔ Social media algorithms	0.159

### 5.4 Assessment of collinearity in the structural model

In structural equation modeling, assessing collinearity among predictor variables is essential to ensure model accuracy. Collinearity can inflate the variance of regression coefficients, making results unreliable. The Variance Inflation Factor (VIF) is used to gauge collinearity severity; a VIF below 5 is generally acceptable (107). In this model, VIF values for predictors of Fitness-related Health Information Use Behavior range from 1.092 to 2.2, well within the acceptable range, indicating no problematic collinearity. This supports the structural model’s suitability for further analysis, as shown in Table 8.

**Table 8:** Collinearity analysis (VIF).

Path	VIF
Needs for autonomy → Fitness-related health information use behavior	2.2
Needs for competence → Fitness-related health information use behavior	2.104
Needs for relatedness → Fitness-related health information use behavior	1.238
Social media algorithms → Fitness-related health information use behavior	1.092
Source credibility → Fitness-related health information use behavior	1.852

### 5.5 Path coefficient analysis

The path coefficient clarifies theoretical relationships among latent variables, with *p*-values obtained using a 5,000-resample two-tailed bootstrapping method. Results indicate that, except for H6 and H7, all hypotheses are statistically significant at the 0.05 level, underscoring key construct relationships. Following Hair (102) criteria, all path *T*-values exceed 1.96 (two-tailed), validating the hypothesized relationships in the structural model. According to Cohen (108), effect sizes for path coefficients are classified as small (0.10), moderate (0.30), and large (0.50). Path significance testing shows that Needs for Autonomy has a small effect on Fitness-related Health Information Use Behavior with a path coefficient of 0.115 (*p* = 0.017), indicating a minor positive impact. Needs for Competence shows a moderate effect (path coefficient = 0.224, *p* < 0.001). Needs for Relatedness also shows a small effect (path coefficient = 0.114, *p* = 0.001), supporting its role in promoting health information use. Social Media Algorithms, though significant (path coefficient = −0.076, *p* = 0.007), have a minor negative effect, suggesting potential suppression of health information use in certain contexts. Source Credibility has the largest impact on Fitness-related Health Information Use Behavior with a path coefficient of 0.446 (*p* < 0.001), highlighting the critical role of trusted sources in motivating fitness-related health information use behaviors. Therefore, H1, H2, H3, H4, and H5 are supported. Detailed coefficients are in Table 9 and Supplementary Appendix A.

**Table 9:** Path significance testing.

Constructs	Original sample (O)	Sample mean (M)	Standard deviation (STDEV)	*T* statistics (O/STDEV)	*p* values	Significance level
Needs for autonomy → Fitness-related health information use behavior	0.115	0.114	0.048	2.381	0.017	Significant
Needs for autonomy → Fitness-related health information use behavior	0.224	0.225	0.049	4.564	0	Significant
Needs for autonomy → Fitness-related health information use behavior	0.114	0.115	0.034	3.35	0.001	Significant
Social media algorithms → Fitness-related health information use behavior	−0.076	−0.077	0.062	2.682	0.007	Significant
Source credibility → Fitness-related health information use behavior	0.446	0.446	0.062	7.243	0	Significant
Social media algorithms **×** Needs for relatedness → Fitness-related health information use behavior	0.079	0.075	0.039	2.043	0.041	Significant
Social media algorithms **×** Needs for autonomy → Fitness-related health information use behavior	0.024	0.024	0.054	0.44	0.66	Not Significant
Social media algorithms **×** Needs for competence → Fitness-related health information use behavior	−0.07	−0.067	0.057	1.236	0.217	Not Significant

### 5.6 Explanation of *R*^2^

In research, *R*-squared (*R*^2^) is a statistical measure used to assess the fit of a model to the observed data, representing the proportion of variability in the dependent variable explained by the model. In this study, it was found that the *R*-squared for Fitness-related Health Information Use Behavior is 0.575, with an adjusted *R*-squared of 0.569 (Table 10). This indicates that the model successfully explains 57.5% of the variability in health information use behavior, meaning that the independent variables in the model (such as Needs for Autonomy, Competence, Relatedness, Social Media Algorithms, and Source Credibility) account for 57.5% of the variation in the dependent variable (health information use behavior). The adjusted R-squared takes into account the number of independent variables and the sample size in the model, and is therefore typically considered a more accurate estimate of model fit. Overall, the model demonstrates a moderate level (104) of explanatory power for health information use behavior (0.5 < *R*^2^ < 0.75).

**Table 10:** Results of *R*-square.

	*R*-square	*R*-square adjusted
Fitness-related health information use behavior	0.575	0.569

### 5.7 Explanation of *f*^2^ and *Q*^2^

f^2^ is an effect size indicator used to assess the relative impact or importance of an independent variable on an endogenous latent variable. The *f*^2^ value helps understand how much a specific predictor variable contributes to explaining an endogenous variable within the model. According to Hair et al. (109), values of 0.02, 0.15, and 0.35 are considered small, medium, and large effects, respectively. In terms of influencing fitness-related health information use behavior, the credibility of the source (*f*^2^ = 0.252) has the most significant medium effect and is the most important influencing factor. Other factors such as the need for autonomy (*f*^2^ = 0.014), the need for competence (*f*^2^ = 0.056), the need for relatedness (*f*^2^ = 0.025), and social media algorithms (*f*^2^ = 0.012) have relatively weaker impacts, all exhibiting small to very small effects. These results suggest that enhancing Source Credibility might be a key strategy to improve the acceptance and use of fitness-related health information (Table 11).

**Table 11:** Results of *f*-square (Effect size).

	Fitness-related Health Information Use Behavior	Effect Size	Strength
Needs for autonomy	0.014	Small	Weak
Needs for competence	0.056	Small	Weak
Needs for relatedness	0.025	Small	Weak
Social media algorithms	0.012	Very Small	Very Weak
Source credibility	0.252	Medium	Moderate

*Q*^2^ is a model evaluation metric used to measure the model’s predictive capability for the data. *Q*^2^ is derived from the Stone-Geisser test, a result of a cross-validation technique. If *Q*^2^ > 0, it indicates that the model is meaningful, with values greater than 0, 0.25, and 0.5 indicating small, medium, and large predictive accuracy of the PLS path model, respectively (104). The study results show that the *Q*^2^ value for fitness-related health information use behavior is 0.301, indicating that the model performs well in predicting this endogenous variable (Table 12).

**Table 12:** Results of *Q*^2^.

Factor	SSO	SSE	*Q*^2^ (=1-SSE/SSO)
Fitness-related health information use behavior	3,600	2516.071	0.301
Needs for autonomy	3,000	3,000	
Needs for competence	3,000	3,000	
Needs for relatedness	3,000	3,000	
Social media algorithms	3,000	3,000	
Source credibility	3,000	3,000	

### 5.8 Moderation by social media algorithms

Using Bayesian two-tailed sampling (standardized) method, we observed that social media algorithms did not moderate the relationship between Needs for Autonomy (*p* = 0.217, *p* > 0.05) and fitness-related health information use behavior, as well as between Needs for Competence (*p* = 0.66, *p* > 0.05) and fitness-related health information use behavior. However, social media algorithms moderated the relationship between Needs for Relatedness and fitness-related health information use behavior (*p* = 0.041, *p* < 0.05), thus validating H8 (Table 8).

## 6 Discussion

The study’s findings reveal significant insights into the motivational and behavioral aspects of fitness-related health information usage among youth on social media, highlighting the impact of intrinsic needs based on Self-Determination Theory—competence, autonomy, and relatedness—as well as external factors such as social media algorithms and source credibility. The need for competence (*f*^2^ = 0.056) and autonomy (*f*^2^ = 0.014) shows that youth are more likely to engage with health information when they feel capable and in control of their fitness journeys. Interestingly, relatedness (*f*^2^ = 0.025) underscores the importance of social connections in motivating health behavior, aligning with findings that peer influence and social support can significantly impact health behaviors (110).

The significant influence of source credibility (*f*^2^ = 0.252) confirms the critical role of trustworthy information sources in health communication effectiveness (111). This underscores that users are more likely to engage with content that they find credible, which is crucial for platforms that aim to influence health behaviors positively. Additionally, the preference and experience of users with social media platforms reveal an important dynamic in the accessibility and consumption of fitness-related health information. TikTok and Red are the most popular social media platforms among youth in China. TikTok enhances the visibility of fitness information by utilizing personalized recommendations and analyzing user behavior data to deliver targeted content (112). In contrast, Red has an advantage in content community and user interaction, and its success stems from close interaction with content creators and rich community content (113). To maximize the impact of fitness information, fitness influencers, app developers, and public health institutions can adopt strategies. Fitness influencers can create engaging tutorial series that build skill step-by-step and actively interact with followers through Q&As in comments, fostering a sense of community and trust. App developers might integrate personalized recommendations to deliver relevant content while featuring a user-sharing space that encourages community interaction. Public health institutions could leverage short, evidence-based video content and partner with influencers to reach wider audiences with reliable health information. These approaches harness platform-specific interactivity and recommendation systems, enhancing both reach and user engagement in fitness content dissemination.

Moreover, the results suggest a potential link between socio-demographic variables and fitness-related health information behavior. Primarily, the participants were youth (average age 22.80) who preferred short-video platforms like TikTok and Red (33.17 and 31.00%, respectively). This age group’s preference for highly interactive and visually engaging content highlights their specific demand for platforms with immediate feedback and entertainment value. Social media platforms could thus enhance video features to better attract this demographic. Additionally, 57.34% of participants were short-term users (under 6 months), indicating a phase-based interest in fitness information: initial enthusiasm often declines over time as familiarity grows. This trend suggests that novice users may seek basic content, whereas long-term users prefer advanced guidance. Platforms may benefit from dynamic content recommendations that adapt to users’ engagement levels, encouraging sustained interest in fitness information.

However, unlike studies that emphasize the overwhelming impact of algorithms on user autonomy (114), this study suggests that algorithms do not significantly diminish autonomy but do modulate the effect of relatedness on health information behavior. These results may be due to factors like information overload and content diversity. Research shows that algorithmic recommendations on social media can lead to information overload, making it challenging for users to filter and focus on fitness-related information, thus weakening the algorithm’s moderating effect (115). Additionally, the wide variety of content on social media can impact the relationship between SDT factors and behavior. With fitness information often mixed with entertainment and fashion content, users may find it hard to maintain focus on fitness topics (116). This suggests that while algorithms increase content visibility, their effectiveness is limited by users’ cognitive load and content variety. Subsequent research could examine how personalized algorithms prioritize content types and optimize recommendations to better meet users’ needs, especially in promoting sustained engagement with fitness information. Also, the unexpected modest impact of autonomy and competence compared to relatedness invites further exploration into the contextual factors that might influence these dynamics.

## 7 Implications and future research

This study offers valuable insights into health communication strategies tailored specifically for youth on social media, enriching both theoretical frameworks and practical applications. Theoretically, this study merges SDT with social media dynamics, enhancing our theoretical understanding of youth’ intrinsic needs and their interactions with external factors like algorithms and source credibility. This foundational approach encourages further exploration of online behaviors in younger demographics. Practically, the findings emphasize the importance for health practitioners and content creators to tailor strategies that align with youth’ distinct preferences and enhance their empowerment and connection on social media. For Chinese youth specifically, this study highlights that their fitness-related health behaviors can be positively shaped through personalized, credible content. Health practitioners and content creators are encouraged to develop strategies that align with youth-specific motivations, such as their need for social connections and reliable information sources. This approach can empower young people in China to adopt consistent and healthy fitness practices, supported by the interactive and algorithm-driven features of popular platforms like TikTok and Red, which resonate strongly with this demographic.

Furthermore, by leveraging these findings, health communication strategies can be optimized to build trust and engagement, ensuring that fitness-related content not only attracts but sustains youth interest in fitness habits over time.

Future studies should address this research’s limitations, conducted solely in China with a culturally diverse young audience. Exploring cross-cultural differences is crucial for tailoring effective health communication on social media globally. Additionally, factors related to social media algorithms can be broken down, e.g., algorithm transparency and user control, which can offer other insights into their impact on youth users’ needs for autonomy. The voluntary sampling method may introduce bias, suggesting that future studies use random sampling or diverse recruitment channels for better representativeness.

As these findings are based on a Chinese cultural context, they may be shaped by collectivist tendencies. For instance, Chinese users are more likely to consider others’ comments and popular trends when selecting fitness content, making social interaction a significant factor in content choice (10). Additionally, local platforms like Red may better align with Chinese users’ preferences, while Western users often prefer platforms like Instagram (9). These cultural differences suggest that interpretations should consider the influence of culture on user behavior, and future cross-cultural studies could further explore fitness information usage across different cultural settings.

## 8 Conclusion

This study set out to explore the intrinsic and extrinsic factors influencing Chinese youth’s use of fitness-related health information on social media, with a focus on the roles of competence, autonomy, relatedness, social media algorithms, and source credibility. The results confirmed that the intrinsic needs of competence, autonomy, and relatedness significantly promote engagement with fitness-related health content, validating the application of SDT within the digital fitness context. Social media algorithms, particularly their personalization and engagement features, were shown to enhance relatedness by connecting users with like-minded communities, yet their impact on autonomy and competence was more complex, potentially moderated by the overload and diversity of content. Additionally, source credibility emerged as a key factor, indicating that trustworthy, expert-driven fitness content is crucial for sustained engagement. By meeting these objectives, this study provides a foundational understanding of the motivational dynamics at play, offering practical insights for health communication strategies targeting Chinese youth.

## Data Availability

The data supporting the findings of this study are available in the Supplementary Material associated with this article. The data are subject to restrictions under the terms of the participant consent forms and cannot be used for commercial purposes.
