# Supplementary figures and images for: What drives Chinese youth to use fitness-related health information on social media? An analysis of intrinsic needs, social media algorithms, and source credibility
Source: Front Public Health. 2024 Dec 5;12:1445778. doi: 10.3389/fpubh.2024.1445778 (PMC11655457; doi:10.3389/fpubh.2024.1445778)

**Appendix A. Structureal model path coefficients loading with T-value.**


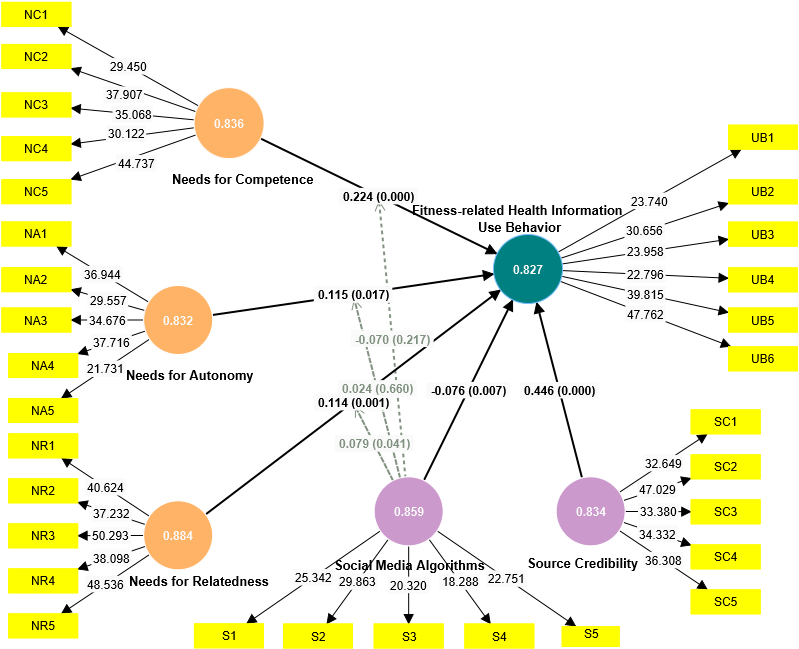

Supplement: Supplementary file 2 [file Table_1.docx]
