# Supplementary material for: What drives Chinese youth to use fitness-related health information on social media? An analysis of intrinsic needs, social media algorithms, and source credibility
Source: Front Public Health. 2024 Dec 5;12:1445778. doi: 10.3389/fpubh.2024.1445778 (PMC11655457; doi:10.3389/fpubh.2024.1445778)
Supplement: Supplementary file 3 [file Table_2.docx]

**Appendix B. Measurements**

| **Constructs** | **Items** |
| --- | --- |
| ***Need for Competence*** | - 1. Using fitness-related health information on social media allows me to acquire skills and knowledge.   2. Using fitness-related health information on social media enhances my overall ability to manage my body.   3. Fitness-related health information on social media provides me opportunities to gain fitness experience.   4. I tend to use fitness-related health information on social media that I am capable of understanding and mastering.   5. When the opportunity arises, I will use fitness-related health information on social media to assess my grasp of this knowledge, experience, and skills. |
| ***Needs for Autonomy*** | 1. I want to be able to decide when and where to use fitness-related health information on social media. 2. I want to choose whether to adopt the advice from any piece of fitness-related health information on social media. 3. I can freely choose the social media platforms to use fitness-related health information. 4. When using fitness-related health information on social media, I need the freedom to express my thoughts and opinions. 5. Despite social media offering a variety of fitness lifestyle templates, I still need the freedom to choose my own lifestyle. |
| ***Needs for Relatedness*** | 1. When using fitness-related health information on social media, I need to social. 2. If I frequently use fitness-related health information on social media, I hope to find one or a group of friends to discuss such information. 3. When using fitness-related health information on social media, I need to develop friendships with those I regularly exchange fitness-related information. 4. If strangers like a comment I leave on a fitness post on social media, it encourages me to use more fitness-related health information. 5. If I frequently message online friends on social media to discuss fitness-related health information, this would encourage me to use more of such information. 6. If my family or friends support my use of fitness-related health information on social media, it encourages me to continue using it. |
| ***Social Media Algorithms*** | 1. If social media algorithms recommend fitness-related health information I am interested in, I will use it more. 2. If social media algorithms filter out fitness-related health information I am not interested in, I will use it more. 3. If social media algorithms personalize fitness-related health information services based on my habits, I will use it more. 4. If I can understand the process of how social media algorithms recommend fitness-related health information to me, I will use it more. 5. If social media algorithms optimize the feedback mechanisms (like likes, comments, shares) for fitness-related health information on the platform, I will use it more. |
| ***Source Credibility*** | 1. If fitness-related health information on social media is fair and ethically considered, I will use it. 2. If fitness-related health information on social media is objective, I will use it. 3. If fitness-related health information on social media comes from authoritative or professional sources, I will directly use it. 4. If fitness-related health information on social media is inspiring, I will use it. 5. If fitness-related health information on social media is logically sound and flows well, I will use it. |
| ***Fitness-related Health Information Use Behavior*** | 1. I have used fitness-related health information on social media in the past six months. 2. If I can search for a wide range of fitness-related topics on social media, I tend to use it to continue obtaining fitness-related health information. 3. I actively use fitness-related health information on social media as needed. 4. I use fitness health information across different social media platforms. 5. If many people are following a particular piece of fitness-related health information, I tend to use it. 6. If fitness-related health information on social media is useful, I will continue to use it in the future. |
